# The interplay between HIF-1α and noncoding RNAs in cancer

**DOI:** 10.1186/s13046-020-1535-y

**Published:** 2020-02-03

**Authors:** Xiafeng Peng, Han Gao, Rui Xu, Huiyu Wang, Jie Mei, Chaoying Liu

**Affiliations:** 1 Department of Oncology, Wuxi People’s Hospital Affiliated to Nanjing Medical University, 299 Qingyang Road, Wuxi, 214023 China; 2 The First Clinical Medicine School, Nanjing Medical University, Nanjing, 211166 China; 3 Wuxi School of Medicine, Jiangnan University, Wuxi, 214122 China; 4 School of Basic Medical Sciences, Nanjing Medical University, Nanjing, 211166 China

**Keywords:** HIF-1α, ncRNA, carcinogenesis, clinical practice

## Abstract

Hypoxia is a classic characteristic of the tumor microenvironment with a significant impact on cancer progression and therapeutic response. Hypoxia-inducible factor-1 alpha (HIF-1α), the most important transcriptional regulator in the response to hypoxia, has been demonstrated to significantly modulate hypoxic gene expression and signaling transduction networks. In past few decades, growing numbers of studies have revealed the importance of noncoding RNAs (ncRNAs) in hypoxic tumor regions. These hypoxia-responsive ncRNAs (HRNs) play pivotal roles in regulating hypoxic gene expression at the transcriptional, posttranscriptional, translational and posttranslational levels. In addition, as a significant gene expression regulator, ncRNAs exhibit promising roles in regulating HIF-1α expression at multiple levels. In this review, we briefly elucidate the reciprocal regulation between HIF-1α and ncRNAs, as well as their effect on cancer cell behaviors. We also try to summarize the complex feedback loop existing between these two components. Moreover, we evaluated the biomarker potential of HRNs for the diagnosis and prognosis of cancer, as well as the potential clinical utility of shared regulatory mechanisms between HIF-1α and ncRNAs in cancer treatment, providing novel insights into tumorigenicity, which may lead to innovative clinical applications.

## Background

Hypoxia is a common hallmark in the tumor microenvironment, and its occurrence originates from an imbalance in the supply and consumption of oxygen by rapidly growing tumors [1, 2]. Intratumoral hypoxic conditions stimulate genetic programs that facilitate cellular adaptations to this environmental pressure, subsequently conferring more aggressive phenotypes to cancer cells, such as altered metabolism, augmented survival, invasion, migration, angiogenesis, and resistance to ionizing radiation and various chemotherapies [3–5]. Among the various transcription factors participating in the regulation of tumor cell fate, hypoxia-inducible factor-1 alpha (HIF-1α), the most important transcriptional regulator in response to hypoxia, has been robustly demonstrated to extensively modulate hypoxic gene expression and the signaling transduction networks related to the aforementioned adaptations [6, 7].

**Fig. 1:**
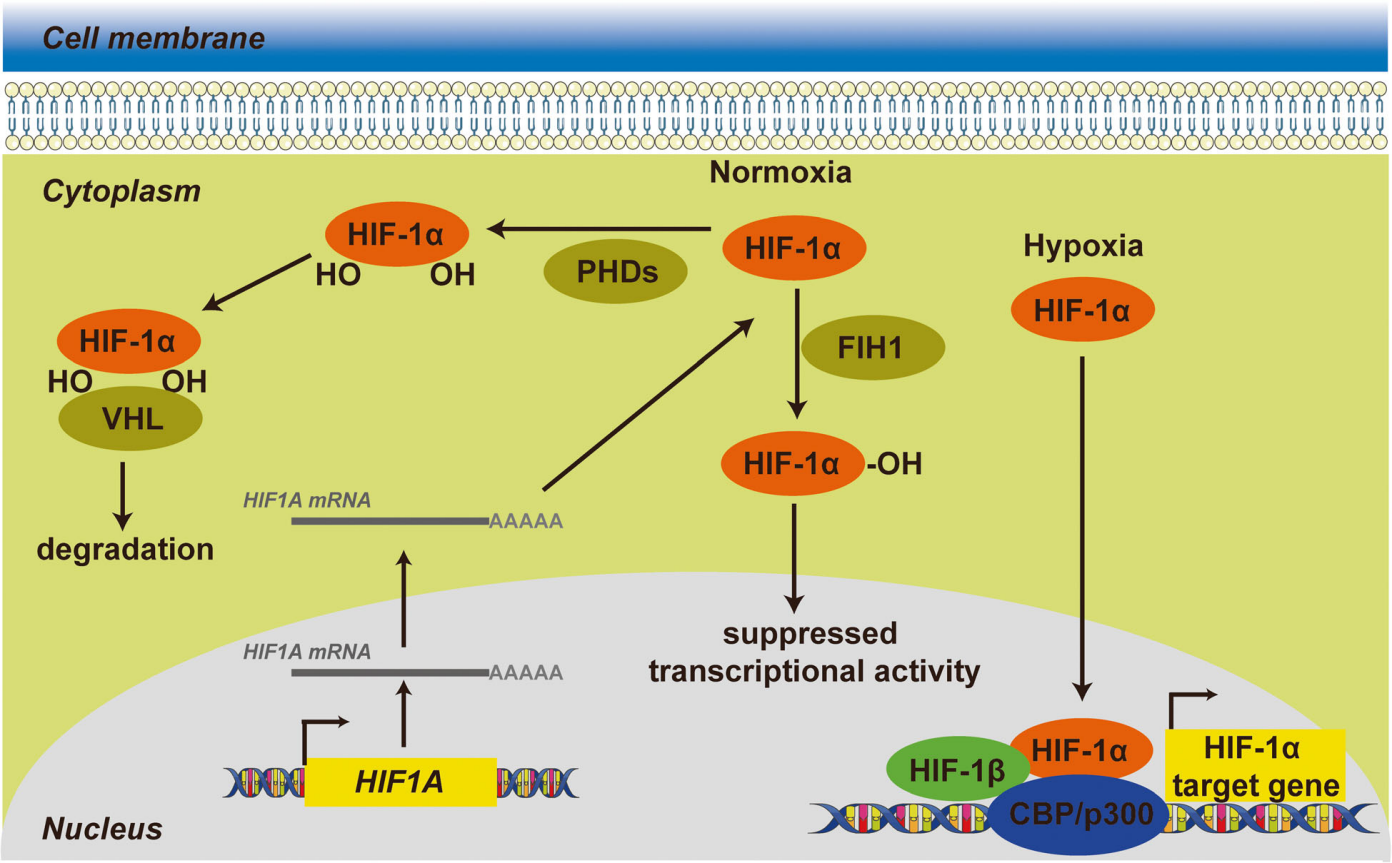
HIF-1α transcriptionally activates target genes in response to hypoxia. Under normoxia, HIF-1α is subjected to hydroxylation by PHDs and other prolyl hydroxylases. Hydroxylated HIF-1α is recognized by VHL proteins that target HIF-1α for subsequent ubiquitination and proteasomal degradation. In addition to regulation of the degradation of HIF-1α, the transcriptional activity of HIF-1α is regulated FIH1, which hydroxylates an asparagine residue of HIF-1α in its C-terminal transactivation domain and therefore blocks the interaction between HIF-1α and CBP/p300. During hypoxia, the hydroxylation reactions are diminished, resulting in HIF-1α accumulation and enhanced transcriptional activity, dimerization with HIF-1β, binding to target genes and activation of target genes through recruitment of CBP/p300 and formation of the transcription initiation complex.

Under normoxic conditions, the conserved proline residues 402 and 564 of HIF-1α are hydroxylated by prolyl hydroxylase domain enzymes (PHDs) that utilize O_2_ [8]. Thereafter, the von Hippel Lindau (VHL) tumor suppressor functions as an E3 ubiquitin ligase to mediate the ubiquitination of HIF-1α by specifically binding to these two prolyl-hydroxylated residues, eventually leading to rapid proteasomal degradation of HIF-1α protein [9, 10]. In addition to the regulation of the degradation of HIF-1α, the transcriptional activity of HIF-1α is also regulated by another asparaginyl hydroxylase, hypoxia-inducible factor 1, alpha subunit inhibitor (FIH1), which hydroxylates an asparagine residue of HIF-1α in its C-terminal transactivation domain [11, 12] and therefore blocks the combination of HIF with the transcriptional coactivator CBP/p300, eventually inhibiting HIF-1α transcriptional activation [13] (Fig. 1). In hypoxic conditions, oxygen deprivation halts the oxygen-dependent hydroxylation activity of PHDs and FIH to elicit the stabilization of HIF-1α, further enabling HIF-1α to translocate to the nucleus and complex with HIF-1β and the transcriptional coactivator CBP/p300 to recognize hypoxia-response elements (HREs) in the promoters of target genes for subsequent transcription [14, 15] (Fig. 1).

Noncoding RNAs (ncRNAs) are a group of RNAs that occupy more than 95% of the human transcriptome without the capacity to encode proteins [16]. Specifically, ncRNAs can be categorized into two groups, small ncRNAs and long ncRNAs (lncRNAs), according to their length, with a cut-off at 200 nucleotides. Due to their lack of protein-coding capacity, ncRNAs have been regarded as transcriptional “noise” for some time. However, a growing number of studies have demonstrated that ncRNAs have critical biological effects on both physiological and pathological processes [17, 18], especially in the field of cancer research [19, 20].

To date, current studies of ncRNAs have mainly concentrated on microRNAs (miRNAs), lncRNAs, and circular RNAs (circRNAs). MiRNAs, which are approximately 20 to 24 nucleotides in length, are a well-known group of small ncRNAs that epigenetically or posttranscriptionally regulate the expression of target mRNAs by imperfectly base pairing with the mRNA 3’-untranslated region (3’-UTR) of target mRNAs. LncRNAs, which have transcripts of more than 200 nucleotides in length, exhibit multiple functions in regulating gene expression through chromatin modification and transcriptional and posttranscriptional regulation [21–23]. Although circRNAs belong to the lncRNA family, researchers tend to discuss them separately, distinguishing them from lncRNAs due to their unique structure.

More than one thousand target genes have been reported to be regulated by HIF-1α to mediate the phenotypes induced by hypoxia [24, 25]. Among these, ncRNAs modulated by hypoxia signaling, which are termed hypoxia-responsive ncRNAs (HRNs), are especially noteworthy, and there are emerging studies concentrated on the exploration of crosstalk between ncRNAs and HIF-1α in multiple tumorigenesis processes. In this review, we briefly elucidate the reciprocal regulation between HIF-1α and ncRNAs in terms of transcription, translation, and protein stability, as well as their effect on the various biological behaviors of tumor cells. In addition, we also attempt to summarize the variegated feedback loop existing in these two components, which is different from simple unidirectional regulation. Finally, we evaluate the potential of prospective HRN biomarkers for the diagnosis and prognosis of cancer, as well as the potential clinical application of regulatory mechanisms shared between HIF-1α and ncRNAs in cancer treatment.

## Regulation of ncRNAs by HIF-1α

Given the relevance of HIF pathways to tumor pathogenesis and the pivotal roles of ncRNAs in gene expression, it is not surprising that substantial effort has been directed toward defining the transcriptional output of ncRNAs in hypoxia-associated malignant progression in the past few years. According to their interplay with the HIF complex, HRNs can be categorized into participating in HIF-1α-mediated direct regulation and HIF-1α-mediated indirect regulation. It is well appreciated that the HIF complex is a crucial transcription factor coordinating the cellular transcriptional response under hypoxic stress [26].

In HIF-1α-mediated direct regulation, HIF-1α directly regulates ncRNAs at the transcriptional level through HREs, which usually reside within the promoter regions of the ncRNAs [27]. In addition, several studies have described hypoxic induction of lncRNAs without the direct involvement of HIF on their promoters. These indirect regulations seem to be achieved through epigenetic mechanisms. Not surprisingly, as an integral hypoxic transcription factor, the HIF complex transactivates the expression of multiple genes, including those involved in epigenetic modifications by histone deacetylases (HDACs) [26]. As a novel hotspot of the ncRNA field, hypoxia-responsive circRNAs (HRCs) have been shown to be of great importance. However, unlike miRNAs and lncRNAs, the mechanisms of HIF-1α-mediated HRC expression are not fully understood [28]. Similarly, several studies uncovered that HIF-1α can directly regulate circRNAs at the transcriptional level through HREs, but more mechanisms have not been reported [29].

### Regulation of miRNAs by HIF-1α

Recently, the number of HRNs that have been identified is expanding rapidly, illustrating the complexity of hypoxia-responsive gene reprogramming and the importance of reconsidering the involvement of the noncoding genome in this adaption [30, 31]. MiRNAs are the most studied subgroup of ncRNAs, and hypoxia-responsive miRNAs (HRMs) have exhibited promising oncogenic and/or tumor-suppressive functions in the oncogenesis and development of cancers [32]. In this section, we systematically discuss the regulatory mechanism of ncRNAs by HIF-1α. As a result, we summarize the functions of miR-210 in tumors in Table 1 as well as other HRMs and their roles in Table 2.

**Table 1:** Summarization of the roles and functions of miR-210 in human cancers

Role in cancer	Cancer types	Targets	Functions	References
oncogene	ovarian cancer	PTPN1	promotes proliferation, inhibits apoptosis	[33]
oncogene	hepatocellular cancer	AIFM3	promotes proliferation, inhibits apoptosis and radiosensitivity	[34]
oncogene	lung cancer	FGFRL1, E2F3, VMP1, RAD52, SDHD	promotes angiogenesis	[35]
oncogene	colon cancer	Bcl-2	enhances autophagy and reduces radiosensitivity	[36]
oncogene	breast cancer	N.A.	promotes proliferation, invasion and migration	[37]
oncogene	hepatocellular cancer	VMP1	promotes migration and invasion	[38]
oncogene	prostate cancer	TNIP1, SOCS1	promotes EMT, invasion and migration	[39]
oncogene	glioma	MNT	promotes hypoxic glioma stem cells stemness and radioresistance	[40]
oncogene	breast cancer	N.A.	N.A.	[41]
oncogene	renal cancer	ISCU1/2	N.A.	[42]
oncogene	gastric cancer	HOXA9	inhibits EMT, invasion and metastasis	[43]
oncogene	breast cancer	N.A.	promotes survival and invasion	[44]
tumor suppressor	esophageal cancer	FGFRL1	inhibits survival and proliferation	[45]
tumor suppressor	laryngocarcinoma	FGFRL1	inhibits proliferation	[46]
tumor suppressor	esophageal cancer	N.A.	promotes differentiation, inhibits radioresistance	[47]
tumor suppressor	renal cancer	ISCU	N.A.	[48]
tumor suppressor	renal cancer	E2F3	inhibits proliferation, induces multinucleation	[49]
tumor suppressor	neuroblastoma	Bcl-2	promotes apoptosis	[50]

**Table 2:** List of hypoxia-responsive miRNAs excepting miR-210

MiRNAs	Cancer types	Status upon hypoxia	Regulatory mechanisms	Targets	Functions	References
miR-21	lung cancer	upregulation	transcriptional activation	PTEN	promotes colony formation, invasion and migration	[51]
miR-382	gastric cancer	upregulation	transcriptional activation	PTEN	promotes proliferation, migration and angiogenesis	[52]
miR-224	gastric cancer	upregulation	transcriptional activation	RASSF8	promotes growth, migration and invasion	[53]
miR-215	glioblastoma	upregulation	transcriptinal proccessing *via* Drosha	KDM1B	promotes growth and neurospheres formation	[54]
miR-494	lung cancer	upregulation	transcriptional activation	PTEN	promotes migration	[55]
miR-145	bladder cancer	upregulation	transcriptional activation	N.A.	promotes apoptosis	[56]
miR-191	breast cancer	upregulation	transcriptional activation	TGFβ2, HuR	promotes proliferation, migration and survival	[57]
miR-27a	gastric cancer	upregulation	transcriptional activation	N.A.	promotes proliferation, survival, multidrug resistance	[58]
miR-424	breast cancer	upregulation	transcriptional activation	PDCD4	inhibits apoptosis and sensitivity to chemotherapy	[59]
miR-21	pancreatic cancer	upregulation	transcriptional activation	N.A.	promotes proliferation, inhibits apoptosis	[60]
miR-185	pancreatic cancer	upregulation	transcriptional activation	N.A.	N.A	[61]
miR-210-3p	oral carcinoma	upregulation	transcriptional activation	RGMA	promotes proliferation	[62]
miR-21	oral carcinoma	upregulation	transcriptional activation	N.A.	promotes migration and invasion	[63]
miR-107	gastric cancer	upregulation	N.A.	N.A.	N.A.	[64]
miR-204	hepatocellular cancer	downregulation	N.A.	VASP	inhibits EMT, migration and invasion	[65]
miR-34a	colorectal cancer	downregulation	transcriptional inhibition	PPP1R11	inhibits EMT, invasion and migration	[66]
miR-548an	pancreatic cancer	downregulation	transcriptional suppression *via* HDAC1	Vimentin	inhibits the proliferation and invasion	[67]
miR-200b	pan-cancer	downregulation	transcriptinal proccessing *via* Dicer	ZEB1/2	inhibits EMT and invasion	[68]
miR-33a	hepatocellular cancer	downregulation	N.A.	Twsit1	inhibits EMT and invasion	[69]
miR-205	prostate cancer	downregulation	transcriptional inhibition	ZEB1/2	inhibits EMT, motility, stemness and cancer-associated fibroblasts reactivity	[70]
miR-101	prostate cancer	downregulation	transcriptional inhibition	EZH2	inhibits invasion, migration, proliferation	[71]
miR-224-3p	glioblastoma, astrocytoma	downregulation	N.A.	ATG5	inhibits mobility, promotes chemosensitivity	[72]
miR-15a	lung cancer	downregulation	N.A.	N.A.	N.A.	[73]

#### Regulation of miR-210 expression by HIF-1α

Among all the miRNAs regulated by hypoxia through HIF-1α signaling, the most studied HRM is miR-210 [74–76]. Numerous studies aiming at the multifarious genes targeted by HIF-1α-induced miR-210 overexpression have highlighted the broad involvement of this mechanism in intricate cancer pathologies, including proliferation [33, 45, 46], apoptosis [34], angiogenesis [35], autophagy [36], metastasis [37–39], and radioresistance [40, 47].

Most studies have uncovered the oncogenic role of miR-210 in human cancers. For instance, given that miR-210 expression can be altered by the HIF-1α/VHL transcriptional system and the inverse correlation of miR-210 expression with outcome variables as an independent prognostic marker in breast cancer [41] and clear cell renal cell cancer [42], Yu *et al.* further hypothesized and identified that miR-210 mediated HIF-1α-induced epithelial-mesenchymal transition (EMT) to drive invasion, recurrence, and metastasis of gastric cancer by regulating the expression of homeobox A9 (HOXA9), a transcription factor which may regulate gene expression, morphogenesis, and differentiation [43]. In both ovarian cancer specimens and cell lines under hypoxic conditions, increasing miR-210 expression shows a positive correlation with HIF-1α overexpression and indicates more aggressive and anti-apoptotic outcomes characterized by a higher tumor stage, larger postoperative residual tumor size, augmented cell proliferation and clone generation. This oncogenic effect of miR-210 in vivo is dependent on the inhibition of protein tyrosine phosphatase, non-receptor type 1 (PTPN1) [33]. Moreover, there is an interesting phenomenon in which increasing the release of exosomes containing miR-210 by breast cancer cells promotes their invasion and assists in their survival, which is hypoxically mediated by the HIF-1α oxygen-sensing system [44].

However, McCormick *et al.* [48] found an unexpected relationship between HIF-1α-induced miR-210 expression and favorable clinicopathological factors, such as reduced proliferation, decreased tumor stage and grade, and improved survival, which is in contrast with the disadvantageous outcomes in clear cell renal cell cancer [42, 49]. Similarly, in neuroblastoma cells, HIF-1α-dependent induction of miR-210 triggered by oxygen/glucose deprivation has been demonstrated to target the 3’UTR of B-cell CLL/lymphoma 2 (Bcl-2) and sequentially promote hypoxia-induced neural apoptosis [50]. Collectively, the role of HIF-1α-induced miR-210 has different roles in various cancers, which need to be further explored to solve this mystery.

#### Regulation of other HRM expression by HIF-1α

Positive regulation of HIF-1α on miRNAs is common in cancer progression. The most likely mechanism is that HIF-1α translocates to the nucleus and forms a complex with HIF-1β and CBP/p300 to recognize the HREs in the promoters of primary miRNAs (pri-miRNAs) for subsequent transcription [77]. The cigarette-smoke-induced malignant transformation of bronchial epithelial cells , including characteristics of amplified colony formation, invasion and migration capacities, is dependent on HIF-1α-induced miR-21 upregulation, which subsequently inhibits phosphatase and tensin homolog (PTEN), a classic tumor suppressor, to activate the Akt/NF-κB pathway [51], while in gastric cancer cells, PTEN inhibition caused by HIF-1α-mediated miR-382 expression inversely restrains the Akt/mTOR signaling pathway, conferring miR-382 with angiogenic effects [52]. Similarly, HIF-1α-induced activation of miR-224 targets Ras association domain family member 8 (RASSF8), stimulating NF-κB transcriptional activity and subcellular distribution to confer gastric cancer with more aggressive phenotypes [53]. An indirect interaction distinguished from the aforementioned direct interaction between HIF-1α and miRNAs is elicited by Drosha, an RNase III enzyme and the key factor for nuclear processing of pri-miRNAs [78]. Specifically, in glioblastoma, HIF-1α promotes miR-215 biogenesis by enhancing the incorporation of pri-miR-215 into the microprocessor Drosha; then, increased miR-215 directly targets the epigenetic regulator lysine (K)-specific demethylase 1B (KDM1B) to enhance adaptation to the hypoxic niche [54].

Not limited to positive regulatory mechanisms, HIF-1α can also negatively regulate miRNA expression mostly in an indirect HIF-1α-mediated manner. In hepatocellular carcinoma, vasodilator-stimulated phosphoprotein (VASP) acts as a tumor premotor and its overexpression at the transcriptional level is mediated by direct binding of HIF-1α to HREs in the VASP promoter region. Moreover, miR-204 is inhibited by HIF-1α to upregulate VASP at the posttranscriptional level, providing a typical instance in which HIF-1α and suppressed miRNAs synergistically regulate the same gene in different ways. However, the reason why HIF-1α negatively regulates miR-204 expression is not clear [65]. Li *et al.* demonstrated that downregulated miR-34a was necessary for hypoxia-induced EMT, invasion and migration in colorectal cancer cells. HIF-1α can directly inhibit the expression of miR-34a in p53-defective colorectal cancer cells, whereas the level of miR-34a is increased in p53-proficient colorectal cancer cells under hypoxia [66]. HIF-1α could recruit HDAC1 to the promoter of pri-miR-548an to transcriptionally suppress miR-548an expression, resulting in the upregulation of the EMT marker vimentin, which facilitates the proliferation and invasion of pancreatic cancer cells [67]. Dicer, an RNase III enzyme responsible for cytoplasmic processing of precursor miRNA [79], is frequently interfered with by HIF-1α in an indirect manner [68]. A previous study of breast cancer also found that the HIF-1/2α-dependent EGFR-AGO2 interaction under hypoxic stress probably triggers AGO2-Y393 phosphorylation to inhibit the binding of Dicer to AGO2, which disrupts the formation of the RISC-loading complex required for pre-miRNA processing of tumor-suppressive miRNAs [80].

In addition, the expression of various miRNAs, including miR-33a [69], miR-494 [55], miR-145 [56], miR-191 [57], miR-27a [58], miR-424 [59], miR-205 [70], miR-21 [60], miR-185 [61], miR-101 [71], miR-210-3p [62], miR-224-3p [72], miR-15a [73], miR-21 [63], and miR-107 [64], has been proven to be HIF-1α-dependent in the progression of various cancers. These findings suggest that the HIF-1α-induced oncogenic effect is caused by transcriptional activation of oncogenic HRMs and inhibition of tumor-suppressive miRNAs to some extent.

### Regulation of lncRNAs by HIF-1α

It is well appreciated that the HIF complex is a crucial transcription factor coordinating the cellular transcriptional response under hypoxic stress. According to their interplay with the HIF complex, hypoxia-responsive lncRNAs (HRLs) can be categorized into HIF-dependent and HIF-independent. We summarize the regulatory mechanisms underlying the HIF-1α**-**altered expression of HRLs in Table 3.

**Table 3:** List of hypoxia-responsive lncRNAs

LncRNAs	Cancer types	Status upon hypoxia	Regulatory mechanisms	Functions	References
lncRNA BC005927	gastric cancer	upregulation	transcriptional activation	promotes invasion and metastasis	[81]
lncRNA BX111	pancreatic cancer	upregulation	transcriptional activation	promotes EMT, proliferation, migration and invasion	[82]
lncRNA UCA1	osteosarcoma	upregulation	transcriptional activation	promotes growth	[83]
lncRNA UCA1	bladder cancer	upregulation	transcriptional activation	promotes proliferation, migration and invasion, inhibits apoptosis	[84]
lncRNA FALEC	prostate cancer	upregulation	transcriptional activation	promotes proliferation, migration and invasion	[85]
lncRNA MALAT1	HeLa and HEK-293T cells	upregulation	transcriptional activation	N.A.	[86]
lncRNA ANRIL	osteosarcoma	upregulation	transcriptional activation	promotes invasion, inhibits apoptosis	[87]
lncRNA NUTF2P3-001	pancreatic cancer	upregulation	transcriptional activation	promotes viability, proliferation and invasion	[88]
lncRNA HOTAIR	lung cancer	upregulation	transcriptional activation	promotes proliferation, migration and invasion	[89]
lncRNA HOTAIR	pan-cancer	upregulation	transcriptional activation	N.A.	[90]
lncRNA MEG3	MCF-7 and HEK-293T cells	upregulation	transcriptional activation *via* recruiting CBP/p300	promotes angiogenesis and the spheroid sprouting	[91]
lncRNA H19	glioblastoma	upregulation	transcriptional activation and recruits SP1	promotes migration and invasion	[92]
lncRNA LET	hepatocellular carcinoma	downregulation	histone deacetylation	promotes migration and invasion	[93]

#### Direct regulation of HRL transcription by HIF-1α

Similar to the classical interactive mode between HIF-1α and miRNAs, HIF-1α can also directly interact with the HREs in the lncRNA BC005927 promoter region, inducing lncRNA BC005927 to play its oncogenic role in gastric cancer by upregulating EPH receptor B4 (EPHB4) [81]. In addition, HIF-1α-mediated direct interactions regulate the expression of numerous lncRNAs, including lncRNA BX111 [82], lncRNA UCA1 [83, 84], lncRNA FALEC [85], lncRNA MALAT1 [86], lncRNA ANRIL [87], and lncRNA NUTF2P3-001 [88], all of which play key roles in the development of tumors. Knowing of the existence of a direct interaction of HIF-1α and the HRE region existing in the lncRNA HOTAIR promoter in non-small-cell lung cancer [89], Bhan *et al.* argued that synchronously with this interaction, MLL1 and CBP/p300 are recruited to the lncRNA HOTAIR promoter region, cooperating with HIF-1α to evoke the HOTAIR gene and promote tumorigenesis [90].

#### Indirect regulation of HRL transcription by HIF-1α

Due to the inability of researchers to identify a HIF-1α binding motif in the MEG3 core promoter, Ruan *et al.* speculated that HIF-1α activated lncRNA MEG3 in an indirect manner in human umbilical vein endothelial cells, in which CBP/p300 recruitment for cAMP responsive element binding protein 1 (CREB) transcriptional activation is also required [91]. Although HIF-1α itself can promote lncRNA H19 expression by interacting physically, the knowledge of a concurrent activation pathway of lncRNA H19 expression depending on the interaction between HIF-1α-induced SP1 and the H19 promoter in aggressive glioblastoma cells further expands existing understanding [92]. In addition, HIF-1α-induced lncRNA expression regulation can be implemented by HDAC3. lncRNA LET is repressed by HDAC3 and contributes to hypoxia-mediated hepatocellular carcinoma metastasis [93].

### Regulation of circRNAs by HIF-1α

Although belonging to the lncRNA family, circRNAs are always discussed separately due to their unique structure with a covalently closed continuous loop. In an experiment on breast cancer cells in a hypoxic environment, researchers found that circZNF292, circDENND4C, and circSRSF4 were upregulated after hypoxia treatment, while among these, only circDENND4C was demonstrated to be activated by the induction of HIF-1α [94]. CircDENND2A was predicted to be an HRC in glioma *via* bioinformatic analysis. Hypoxia-induced overexpression of circDENND2A promotes the migration and invasion of glioma cells by sponging miR-625-5p [95]. In addition, more HRCs, including circRNA_403658, circDENND4C, and circRNA_0000977, have been identified to participate in cancer progression by sponging corresponding miRNAs [29, 96, 97]. Although limited research has uncovered the role of HRCs, promising functions of circRNAs in human cancers have been preliminarily established, and we believe that HRCs will be the next hotspot in the research field of hypoxia-induced cancer progression.

## Regulation of HIF-1α expression by ncRNAs

To date, most HRNs are functionally characterized as having profound impact on tumorigenesis in a spectrum of cancer types. However, as a type of gene regulator, ncRNAs can participate in regulating gene expression at multiple levels. MiRNAs directly affect HIF-1α expression and activity, while others may have indirect regulations. LncRNAs have diverse regulatory functions, which can modulate chromatin remodeling, transcriptional regulation, posttranscriptional processing, and translation [98, 99]. Emerging reports have suggested the function of lncRNAs as competing endogenous RNAs (ceRNAs) for miRNAs to regulate related mRNA expression at the posttranscriptional level [100], including HIF-1α mRNA. In summary, ncRNAs can mediate HIF-1α at the posttranscriptional level by various mechanisms, which is essential for the regulation of HIF-1α expression. We summarize the regulatory mechanisms of HIF-1α expression by ncRNAs in Table 4.

**Table 4:** ncRNA-mediated regulation of HIF-1α and cancer progression

NcRNAs	Cancer types	Functions	Regulatory mechanisms	Reference
miR-33b	osteosarcoma	inhibits proliferation and migration	post-transcriptional regulation	[101]
miR-338-3p	hepatocellular cancer	inhibits viability and induces apoptosis, enhances the sensitivity to sorafenib	post-transcriptional regulation	[102]
miR-138	ovarian cancer	inhibits migration and invasion	post-transcriptional regulation	[103]
miR-576-3p	glioma	inhibits migration and proangiogenic abilities	post-transcriptional regulation	[104]
miR-18a-5p	lung cancer	promotes radiosensitivity	post-transcriptional regulation	[105]
miR-3662	hepatocellular cancer	inhibits warburg effect and growth	post-transcriptional regulation	[106]
miR-143-5p	gallbladder cancer	inhibits EMT, proliferation, migration and invasion	post-transcriptional regulation	[107]
miR-143	cervical cancer	inhibits proliferation, promotes apoptosis	post-transcriptional regulation	[108]
miR-106a/b	breast cancer	inhibits stem-like cell specific, self-renewal and sphere-forming phenotype	post-transcriptional regulation	[109]
miR-199a-5p	melanoma	inhibits proliferation, induces arrest	post-transcriptional regulation	[110]
miR-20b	osteosarcoma	inhibits proliferation and invasion	post-transcriptional regulation	[111]
miR-18b	melanoma	inhibits proliferation, induces arrest, inhibits the glycolysis	post-transcriptional regulation	[112]
miR-622	lung cancer	inhibits migration and invasion	post-transcriptional regulation	[113]
miR-33a	melanoma	inhibits proliferation, invasion and metastasis	post-transcriptional regulation	[114]
miR-338	nasopharyngeal carcinoma	inhibits migration and proliferation	post-transcriptional regulation	[115]
miR-20b	hepatocellular cancer	inhibits proliferation, inhibits apoptosis	post-transcriptional regulation	[116]
miR-199a-5p	multiple myeloma	inhibits migration, promotes adhesion, inhibits endothelial cells migration	post-transcriptional regulation	[117]
miR-199b	prostate cancer	inhibits growth, promotes death	post-transcriptional regulation	[118]
miR-199a	hepatocellular cancer	inhibits proliferation	post-transcriptional regulation	[119]
miR-138	renal cancer	promotes apoptosis, inhibits migration	post-transcriptional regulation	[120]
miR-22	colon cancer	inhibits endothelial cell growth and invasion	post-transcriptional regulation	[121]
lncRNA LINC00152	gallbladder cancer	promotes migration, invasion and EMT	post-transcriptional regulation	[122]
lncRNA PVT1	lung cancer	promotes viability and proliferation	post-transcriptional regulation	[123]
lncRNA HOTAIR	renal cancer	promotes proliferation, migration and EMT, inhibits apoptosis	post-transcriptional regulation	[124]
lncRNA ROR	hepatocellular cancer	promotes viability	post-transcriptional regulation	[125]
lncRNA NEAT1	osteosarcoma	promotes proliferation and invasion	post-transcriptional regulation	[126]
lncRNA UCA1	acute myelocytic leukemia	promotes glycolysis and chemoresistance	post-transcriptional regulation	[127]
lncRNA PVT1	gastric cancer	promotes proliferation and invasion	post-transcriptional regulation	[128]
lncRNA DANCR	nasopharyngeal carcinoma	promotes invasion and metastasis	post-transcriptional activation *via* ILF3/ ILF2	[129]
circPIP5K1A	lung cancer	promotes proliferation and metastasis	post-transcriptional regulation	[130]
circRNA_0046600	hepatocellular carcinoma	promotes migration	post-transcriptional regulation	[131]
miR-214	lung cancer	promotes invasion, proliferation and migration	transcriptional activation *via* ING4	[132]
miR-206	lung cancer	inhibits proliferation and angiogenesis, promotes apoptosis	transcriptional inhibition *via* 14-3-3ζ/STAT3 axis	[133]
miR-675-5p	glioma	promotes angiogenesis	stabilize the mRNA *via* HuR	[134]
lncRNA CPS1-IT1	hepatocellular cancer	inhibits EMT, proliferation, migration and invasion	transcriptional inhibition *via* Hsp90	[135]
lncRNA PVT1	gastric cancer	promotes survival, inhibits apoptosis	transcriptional activation *via* mTOR	[136]
miR-128	prostate cancer	inhibits growth and metabolism	translational inhibition *via* RPS6KB1	[137]
lncRNA MEG3	lung cancer	inhibits malignant transformation	translational inhibition *via* Akt/p70S6K/S6 aixs	[138]
lncRNA UBE2CP3	hepatocellular cancer	promotes proliferation, migration and angiogenesis	translational activation *via* ERK/p70S6K aixs	[139]
multiple miRNAs	lung cancer	promotes angiogenesis	post-translational activation *via* FIH1	[140]
miR-135b	head and neck squamous cell carcinoma	promotes proliferation, migration, colony formation and angiogenesis	post-translational activation *via* FIH1	[141]
miR-182	lung cancer	promotes glucose metabolism and proliferation	post-translational activation *via* FIH1	[142]
miR-31	colorectal cancer	promotes proliferation, migration and invasion	post-translational activation *via* FIH1	[143]
miR-592	hepatocellular carcinoma	inhibits glycolytic metabolism and proliferation	post-translational inhibition *via* WSB1	[144]
miR-543	osteosarcoma	promotes glycolytic metabolism and proliferation	post-translational activation *via* PRMT9	[145]
miR-183	glioma	N.A.	post-translational activation *via* IDH2/α-KG axis	[146]
miR-23b	glioma	promotes proliferation and migration, inhibits apoptosis	post-translational activation *via* VHL	[147]
miR-145	colorectal cancer	inhibits proliferation, migration and invasion	post-translational inhibition *via* Akt/ERK axis	[148]
miR-30e	breast cancer	inhibits proliferation, migration and invasion	post-translational inhibition *via* IIRS1/Akt/ERK axis	[149]
miR-26a	hepatocellular cancer	inhibits angiogenesis	post-translational inhibition *via* PIK3C2α/Akt axis	[150]
miR-99a	breast cancer	inhibits migration, invasion, sphere formation	post-translational inhibition *via* mTOR signals	[151]
lncRNA ENST00000480739	pancreatic cancer	inhibits invasion	post-translational inhibition *vi*a OS9	[152]
miR-22	chronic myelogenous leukemia	enhances the sensitivity to imatinib	block HIF-1α nuclear transfer *via* IPO7	[153]
lncRNA H19	multiple myelom	promotes hypoxia-induced adhesion on the stroma	promotes HIF-1α nuclear translocation	[154]
lncRNA MIR31HG	oral cancer	promotes proliferation, migration and invasion	facilitates the recruitment of HIF-1 complex	[155]
lncRNA NDRG1-OT1	breast cancer	N.A.	acts as a scaffold for recruiting HIF-1α	[156]

### Posttranscriptional regulation of HIF-1α expression by ncRNAs

MiRNAs play significant regulatory roles in eukaryotes by binding to the 3’-UTRs of corresponding mRNA transcripts, leading to silencing of the target gene at the posttranscriptional level. A large number of studies have confirmed the existence of the direct interplay between miRNAs and the 3’-UTR of HIF-1α [101–121]. Although the classic mechanism is widespread and important in tumors, we do not describe it in detail in the section due to the simplicity of the interaction.

Based on the previous notion that HIF-1α is a target of miR-138 [120], Cai *et al.* proposed that lncRNA LINC00152 functions as an miRNA sponge for miR-138 through a direct interaction to abrogate the suppressive effect of miR-138 on the expression of HIF-1α [122]. Intriguingly, an almost identical role of lncRNA PVT1 acting as ceRNA for miR-199a-5p in non-small-cell lung cancer under hypoxia was later verified [123]. Additionally, the ceRNA roles of lncRNA HOTAIR [124], Linc ROR [125], lncRNA NEAT1 [126], lncRNA UCA1 [127], and lncRNA PVT1 [128] for their respective miRNAs in cancer progression have also been demonstrated. In nasopharyngeal carcinoma, regulation at the posttranscriptional level has been further extended. To be more specific, lncRNA DANCR was found to directly interact with the ILF3/ILF2 complex, and interleukin enhancer binding factor 3 (ILF3), as the most enriched DANCR-binding protein, is a double-stranded RNA-binding protein and can complex with ILF2 to stabilize mRNA and regulate gene expression, subsequently stabilizing the HIF-1α mRNA and leading to nasopharyngeal carcinoma metastasis [129].

Similar to the classic mechanism by which lncRNAs participate in cancer prognosis, the most common mechanism by which circRNAs regulate biological processes is also related to the HIF-1α model. This mechanism mainly involves three kinds of RNAs, including mRNAs, pseudogene transcripts and lncRNAs, but circRNAs have followed lncRNAs in becoming a novel hotspot of research on the ceRNA family. Research conducted by Chi *et al.* suggested that circRNA circPIP5K1A functions as a miR-600 sponge to inhibit miR-600 to disrupt the interaction at the 3’-UTR between HIF-1α and miR-600 to promote HIF-1α posttranscriptional expression, as well as proliferation and metastasis of non-small-cell lung cancer [130]. In addition, in hepatocellular carcinoma, circRNA_0046600 could upregulate HIF-1α by sponging miR-640 to promote cancer progression [131]. CircRNAs are a novel research focus,, so no additional studies on the regulatory roles of circRNAs in HIF-1α expression are currently available. Given the significant role of circRNAs in regulating target gene expression, we speculate that circRNAs should be the next focus in the field of ncRNA-mediated regulation of HIF-1α expression.

### Transcriptional regulation of HIF-1α expression by ncRNAs

In addition to the basic interaction between miRNAs and the 3’-UTR of HIF-1α, miRNA-mediated transcriptional regulation of HIF-1α expression is a common mechanism in cancer progression. MiR-214 upregulates HIF-1α and VEGFA with the suppression of ING4 to promote the invasion, proliferation and migration of non-small-cell lung cancer cells [132], and a possible mechanism is that ING4, which is recruited by egl-9 family hypoxia-inducible factor 1 (EGLN1), unexpectedly has no effect on HIF-1α degeneration but acts as an adapter protein to recruit transcriptional repressors to regulate HIF activity [157]. MiR-206 can attenuate the growth and angiogenesis of non-small-cell lung cancer cells through the 14-3-3**z**/STAT3/HIF-1α/VEGF pathway. In particular, 14-3-3ζ binds to p-STAT3 (Ser727) and increases its activation. Knockdown of STAT3 blocks the 14-3-3ζ-induced increase in HIF-1α mRNA expression and attenuates the 14-3-3ζ-induced binding of HIF-1α to the VEGF promoter [133]. In addition, Dico *et al.* reported that miR-675-5p interacts with the RNA binding protein HuR to stabilize the mRNA of HIF-1α, along with its additional inhibitory effect on VHL [134].

Moreover, at the transcription level of HIF-1α expression, experimental evidence of lncRNA-mediated regulation already exists. Wang *et al.* suggested that lncRNA CPS1-IT1 could serve as an Hsp90 cochaperone, and this interaction in turn reduces the binding affinity between Hsp90 and HIF-1α, leading to transcriptional inactivation of HIF-1α and diminished EMT of hepatocellular carcinoma cells [135]. In addition, the lncRNA-mediated regulation of the mTOR/HIF-1α/P-gp signaling pathway marked by increased HIF-1a mRNA levels in gastric cancer cells might also suggest the alteration of HIF-1α transcriptional activity [136]. Although the function of lncRNAs as transcriptional regulators has been widely explored, the mechanisms underlying these functions remain poorly understood and require further investigation.

### Translational regulation of HIF-1α expression by ncRNAs

MiR-128, which is regulated by snail family zinc finger 1 (SNAIL) transcriptionally, in turn modulates the expression of ribosomal protein S6 kinase, polypeptide 1 (RPS6KB1), also known as p70S6K, and afterwards disrupts downstream HIF-1α at the translational level and consequently suppresses pyruvate kinase 2 (PKM2) expression to inhibit the growth and metabolism of prostate cancer cells [137], which expands the interplay between HIF-1α and miRNA at the translational level.

As for the translational activity of HIF-1α, lncRNA MEG3 was found to be decreased after nickel exposure, which triggers downstream c-Jun/ PH domain and leucine rich repeat protein phosphatase 1 (PHLPP1) to activate the Akt/p70S6K/S6 axis. Enhanced phosphorylation at Ser235/236 of the 40S ribosomal protein S6 therefore boosts HIF-1α translation in the nickel-induced malignant transformation of human bronchial epithelial cells [138]. In hepatocellular carcinoma cells, overexpressed lncRNA UBE2CP3 enhances human umbilical vein endothelial cell proliferation, migration and angiogenesis, which is attributed to the ERK/p70S6K/HIF-1α/VEGFA signaling axis activated by lncRNA expression deviating from normal status [139]. Distinctly, lncRNAs are defined as ncRNAs without translational function. However, during HIF-1α translation, lncRNAs play indispensable roles.

### Posttranslational regulation of HIF-1α expression by ncRNAs

Complexes formed between HIF the coactivators CBP/p300 are essential for HIF transcriptional activation. FIH1, which blocks the interaction between HIF-1α and CBP/p300, is validated to be downregulated because of a corresponding miRNA deficiency in tumors, consequently suppressing the tumor hypoxia response and angiogenesis by suppressing HIF-1α transcription and VEGF production [140]. Similar mechanisms of miR-135b, miR-182, and miR-31 have been confirmed in head and neck squamous cell carcinoma [141], non-small-cell lung cancer [142] and colorectal cancer [143], respectively.

The stability of HIF-1α is a critical factor in its action on relevant gene expression, and WD repeat and SOCS box containing 1 (WSB1) has been reported to enhance the HIF-1α protein stability derived from the abnormally low expression of miR-592 in hepatocellular carcinoma cells with enhanced glycolysis and proliferation [144]. In osteosarcoma cells, which have a high energy demand but low ATP-generating efficiency, increasing miR-543 targets the 3’-UTR of protein arginine methyltransferase 9 (PRMT9) to decrease PRMT9-induced HIF-1α instability; thereafter, elevated HIF-1α boosts glycolysis and proliferation of osteosarcoma cells [145]. As an indispensable molecule in the degradation of HIF-1α, the role of PHD in HIF-1α stabilization should not be ignored. Indeed, Tanaka *et al.* indicated that upregulated miR-183 in glioma was able to inhibit isocitrate dehydrogenase 2 (IDH2) levels, which elevated HIF-1α levels by reducing the cellular levels of α-KG, a substrate of PHD [146]. In glioma, the targeted inhibitory effect of increasing miR-23b on VHL unsurprisingly activates HIF-1α/VEGF signaling to promote tumor progression [147].

Proteasomal degradation is often regulated by phosphorylation [158], and blocked activation of the Akt and ERK1/2 pathways caused by miR-145-mediated N-RAS and insulin receptor substrate 1 (IRS1) expression inhibition was confirmed to suppress the expression of HIF-1α and downstream VEGF in restricted colorectal cancer growth, which is speculated to depend on its interference with the normal HIF-1α protein degradation process [148]; in addition, almost the same signaling initiated by miR-30e can be seen in breast cancer [149]. Analogously, the PIK3C2α/AKT/HIF-1α/VEGFA pathway regulated by miR-26a plays a role in inhibiting angiogenesis in hepatocellular carcinoma [150]. Because of its important role in the PI3K/Akt/mTOR signaling pathway [159], mTOR and downstream HIF-1α have been experimentally suggested to be inhibited by miR-99a, which reverses the breast cancer stem cell malignant phenotype [151].

LncRNAs also play critical roles in the posttranslational regulation of HIF-1α expression. Osteosarcoma amplified 9 (OS9) has an overall effect on the degradation of HIF-1α, including hydroxylation, VHL binding, and proteasomal degradation, by interacting with both HIF-1α and PHDs [160], and lncRNA ENST00000480739 contributes to metastasis and progression of pancreatic ductal adenocarcinoma by targeting and upregulating HIF-1α [152]. Whether other forms of lncRNA-related posttranslational regulation are essential for HIF-1α needs to be further explored.

### Nuclear transfer of HIF-1α mediated by ncRNAs

The nuclear transfer of HIF-1α is also affected by miRNAs. Importin 7 (IPO7) is a mediator specifically related to HIF-1α nuclear translocation [161], while in chronic myelogenous leukemia cells under curcumin treatment, there is a curcumin-induced downregulation of IPO7 expression caused by miR-22 activation, which further elicits blocked cytoplasm-to-nucleus shuttling of HIF-1α to restrain the glycolytic enzyme profile [153].

Similar to miRNAs, lncRNA H19 has been confirmed to positively participate in HIF-1α nuclear translocation to drive multiple myeloma cell dissemination, although the specific molecules responsible for this procedure are unknown [154]. As a transcription factor, HIF-1α plays an essential role in the nucleus. Thus, the regulation of HIF-1α nuclear transfer by ncRNAs is a promising regulatory mechanism to block the oncogenic function of HIF-1α in cancer progression.

### Regulation of HIF-1α activity *via* scaffolding by ncRNAs

The direct interaction between HIF-1α and lncRNAs is not confined to the 3’-UTR. Shih *et al.* have demonstrated an extremely important role of lncRNA MIR31HG, which acts as a co-activator and complexes with HIF-1α to facilitate the recruitment of the HIF-1 complex, augmenting the HIF-1 transcriptional network essential for oral cancer progression and leading to metabolic reprogramming, increased sphere-forming ability and metastasis [155]. However, lncRNA NDRG1-OT1 was reported to act as a scaffold for recruiting HIF-1α *via* its third-quarter fragment, rather than whole molecule, to increase the expression of the downstream gene N-myc downstream regulated gene 1 (NDRG1) in breast cancer cells under hypoxia, along with the different effects of the remaining fragments on the same target gene [156].

## Feedback loops between HIF-1α and ncRNAs

In addition to the unidirectional regulation pattern, emerging studies have found that there are direct and indirect feedback loops between HIF-1α and miRNAs, which are much more complicated than simple one-way effects. Generally, the formation of these feedback loops makes the posttranscriptional regulation between HIF-1α and miRNA more diverse than that of the original linear structure.

### Positive feedback loops between HIF-1α and ncRNAs

#### Positive feedback loops between HIF-1α and miRNAs

Joshi et al*.* revealed that based on the mutual inhibitory relationship in the HIF-1α-DNM2 and HIF-1α-miR-199a interaction, dynamin 2 (DNM2), HIF-1α and miR-199a, which arises from the opposite strand of the DNM2 gene, are integrated into a feedback loop, which increases both the posttranscriptional level and protein stability of HIF-1α to promote ovarian cancer metastasis [162], and reciprocal suppression between miR-20b and HIF-1α at the transcriptional and posttranscriptional levels also plays a role in fine-tuning the adaptation of tumor cells to different oxygen concentrations [163].

Given the decreased expression of miR-126 observed in the tumors of renal cell carcinoma patients who experienced metastasis [164] or recurrence [165], the positive feedback circuit featuring tumorigenic miR-126 deactivation, increased expression of solute carrier family 7, member 5 (SLC7A5) and SEPRINE1, and stimulated mTOR-dependent HIF1/2α translation has been confirmed to advance metastasis and therapeutic resistance in clear cell renal cell cancer [166], which also enriches the understanding of the effects of HIF-1α translation in the feedback pathway.

The stabilization of HIF-1α is also precisely regulated in various molecular processes. Puisse´gur *et al*. described in detail that in A549 lung cancer cells, miR-210 is upregulated by hypoxia-induced HIF-1α; afterward, increased miR-210 represses the electron transport chain *via* succinate dehydrogenase complex, subunit D (SDHD), and consequent accumulation of succinate inhibits PHD to stabilize HIF-1α, thus forming a positive-autoregulatory loop [167]. Based on this feedback enhancement mechanism, the researchers later confirmed that this circular HIF-1α/miR-210 interaction decreases the mortality rate and promotes the radioresistant phenotype of non-small-cell lung carcinoma cell lines [168]. A similar oncogenic hypoxic circuit, in which the role of SDHD is replaced by glycerol-3-phosphate dehydrogenase 1-like (GPD1L), has been shown to be involved in the apoptosis of triple-negative breast cancer cells [169]. Irreversible activation of the HIF-1α-related pathway via stimulation by the initial activation of HIF-1α due to hypoxia and PTEN/PI3K/Akt activation, the HIF-1α-induced overexpression of miR-182, and the resultant limited PHD2 and FIH1 expression due to miR-182 overexpression eventually results in HIF-1α protein accumulation as well, facilitating angiogenesis and tumor growth in prostate cancer [170]. To complicate matters further, there are two positive feedback loops coexisting in multidrug-resistant hepatocellular cancer cells, namely, HIF-1α/miR-183/IDH2/HIF-1α and HIF-1α/miR-183/SOCS6/p-STAT3/HIF-1α, which may affect HIF-1α at the protein stability level [171].

#### Positive feedback loop between HIF-1α and lncRNA

The feedback loop between HIF-1α and lncRNA is also of great concern. Given that lncRNA MALAT1 enhances the disassociation of VHL from HIF-1α to result in the accumulation of HIF-1α and the Warburg effect in human hepatic L-02 cells under arsenite exposure [172], Ikeda *et al.* further revealed that HIF-1α drives a positive feedback loop composed of HIF-1α, KDM3A and lncRNA MALAT1, where the HIF-1α-inducible histone modulator KDM3A promotes lncRNA MALAT1 transcription *via* histone demethylation at the lncRNA MALAT1 promoter, and the resulting increase in lncRNA MALAT1 in turn accelerates the stabilization of HIF-1α to contribute to glycolytic activation of multiple myeloma under a hypoxic microenvironment [173].

HIF-1α translation is also tightly regulated by a feedback loop. Inspired by the function of mTOR to selectively regulate translation of the HIF-1α mRNA transcript [174], as well as the activation effect of lncRNA MALAT1 on mTOR [175], Zhang *et al.* envisioned a MALAT1/mTOR/HIF-1α loop-mediated increase in pro-angiogenic factors in the angiogenesis process of osteosarcoma [176]. The direct interaction between HIF-1α and HREs in lncRNA DARS-AS1 is capable of upregulating the expression of this lncRNA, which resorts to downstream RBM39/mTOR signaling to continuously stimulate the translation of HIF-1α, hence jointly promoting myeloma malignancy [177].

The altered stability of HIF-1α is definitely another important output of the dynamic feedback loop. For example, in previous research on the Warburg effect, Yang *et al.* proclaimed that transcriptionally upregulated lincRNA-p21 (induced by HIF-1α) is able to bind HIF-1α and VHL, therefore blocking the VHL-HIF-1α interaction to elicit HIF-1a accumulation for augmented glycolysis [178]. In experiments on aerobic glycolysis in breast cancer cells, Chen *et al.* found that PHD2, rather than VHL, complexes with the special RNA stem-loop structure of lncRNA HISLA derived from the extracellular vesicle transmission of tumor-associated macrophages, which interferes with its own binding to HIF-1α and prevents HIF-1α from being hydroxylated and degraded. The resulting enhancement of glycolysis and accumulation of lactate caused by HIF-1α activation stimulates lncRNA HISLA transcription in macrophages *via* ERK/ELK1 signaling in turn [179].

### Negative feedback loop between HIF-1α and ncRNA

In addition to the positive feedback loop that causes continuous activation of pathway components, a negative feedback loop between HIF-1α and ncRNAs leading to the restriction of molecular members has also been confirmed by some researchers. In human umbilical vein endothelial cells, there is a negative regulatory loop containing miR-439 and HIF-1α in which HIF-1α induces miR-439 to bind to and destabilize HIF-1α mRNA, hence reducing the activity of HIF-1α in turn. Moreover, confirmation of this mechanism in HeLa cells further exhibited its significance in cancer therapeutics [180]. Similarly, based on this negative loop, in pancreatic cancer, HIF-1α-induced miR-646 expression was shown to target migration and invasion inhibitory protein (MIIP) to inhibit the deacetylation ability of HDAC6, which eventually promoted the acetylation and proteasomal degradation of HIF-1α [181].

**Fig. 2:**
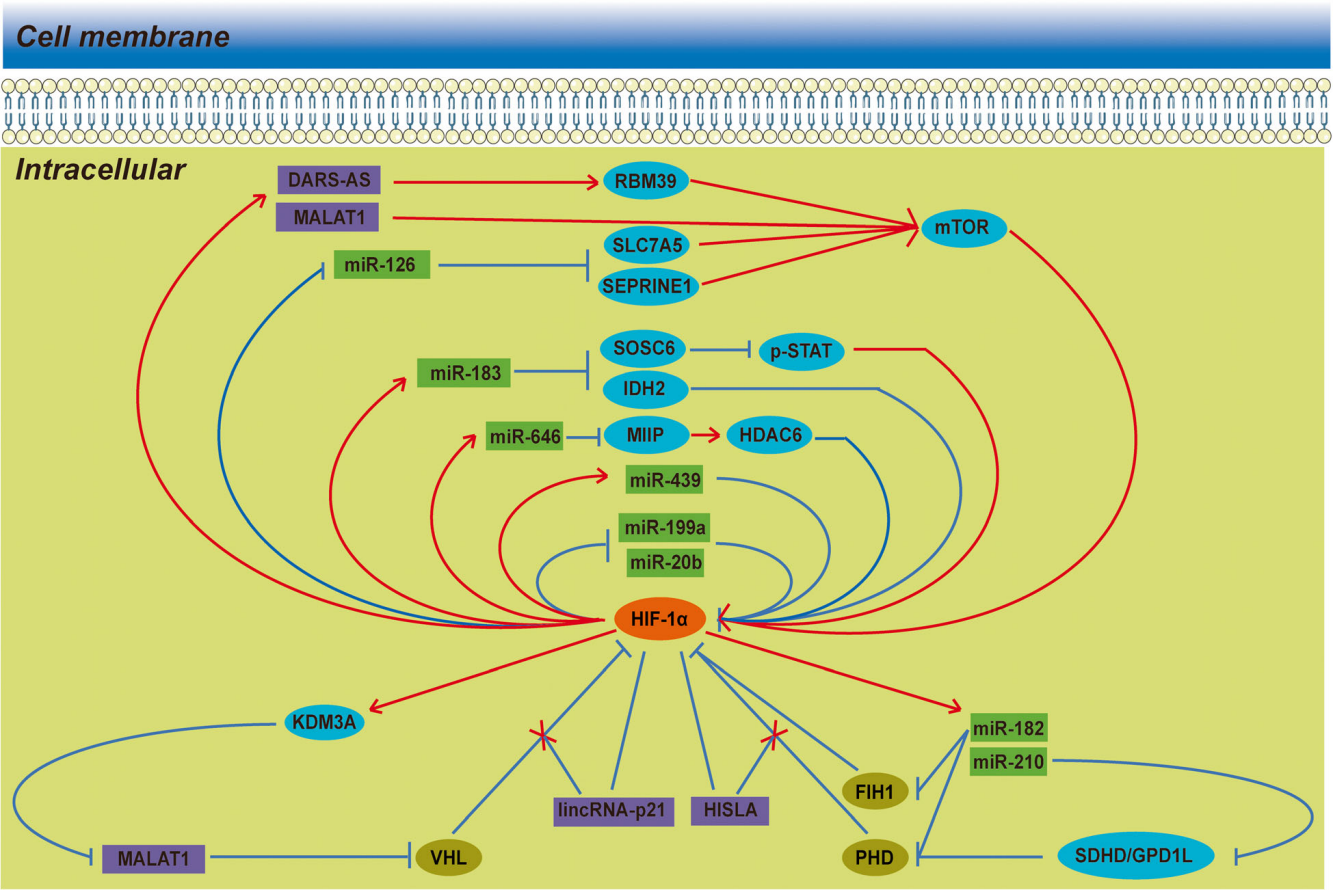
Reciprocal feedback loops between HIF-1α and ncRNAs. In addition to a unidirectional regulation pattern, there are several direct or indirect feedback loops between HIF-1α and ncRNAs. It seems quite feasible that the ncRNAs, HIF-1α and other co-operators would eventually intertwine to form mutually reciprocal feedback loops in both positive and negative manners. In addition to common feedback loops, lincRNA-p21 and HISLA can block VHL- and PHD-dependent HIF-1α repression instead of directly interacting with HIF-1α and other co-operators.

Collectively, it seems quite feasible that ncRNAs, HIF-1α and other co-operators would eventually intertwine to form mutually reciprocal feedback loops in both positive and negative manners. We summarize these reciprocal feedback loops in Fig. 2. In these loops, any alteration in the expression level of any member would disturb the overall balance of the network, resulting in a shift to transcriptional reprogramming, posttranscriptional regulation or translational stability.

## Perspectives on HIF-1α and ncRNAs in clinical practice

### HRNs as potential biomarkers in diagnosis and prognostic evaluation

Several kinds of HRNs have shown unique value in the diagnosis of various tumors. In pancreatic cancer, plasma profiling of four miRNAs, including hypoxia-sensitive miR-210, and determination of their sensitivity and specificity values promises to generate feasible blood-based biomarkers for the early detection of pancreatic cancer [182], while the significantly increased expression of miR-107 seen in both tumor tissues and serum and its correlation with HIF-1α expression suggest the practicality of using miR-107 as a biomarker for the detection of gastric cancer and tumor hypoxia [64]. In colorectal carcinoma, circulating miR-210, miR-21 and miR-126 present high value as noninvasive markers for early diagnosis, screening, and prognosis [183].

HRNs are of great significance in evaluating the prognosis of tumors. In pancreatic cancer, the expression of miR-646 [181] and miR-548 [67] is correlated with clinicopathological indicators such as TNM stage and overall survival (OS), and hypoxia-induced lncRNA NUTF2P3-001 overexpression also indicates advanced TNM stage and shorter survival time of patients [88]. Both Low expression of miR-592 [144] and high expression of miR-130b [184] can bring about poorer OS in hepatocellular carcinoma patients. For gastric cancer, it has been demonstrated that miR-421 regulated by HIF-1α not only causes longer OS, but also can shorten the time to relapse of patients [185], and lncRNA BC005927 induced by hypoxia is also frequently upregulated in gastric cancer samples, showing adverse effects on a series of prognostic parameters, such as TNM stage, lymph node metastasis, and survival time [81]. Not surprisingly, scholars have revealed that aberrant expression of lncRNA H19 [92] and miR-215 [186] in glioblastoma confers a poor prognosis for patients. With regard to triple-negative breast cancers, a type of breast cancer with poor prognosis, patients with relatively low expression of miR-210 fortunately experienced significantly better disease-free and overall survival than those with high expression of miR-210 in a study in Japanese patients [187]. In addition, a strong correlation between high lncRNA EFNA3 expression and shorter metastasis-free survival was found in breast cancer patients [188], undoubtedly enriching the prognostic value of lncRNAs in this prevalent cancer. Innovative extraction and identification of circulating exosomal miR-21 from the serum of patients with oral squamous cell carcinoma and its close affinity with T stage, lymph node metastasis, and HIF-1α expression further supported its prognostic value, as well as the therapeutic value of inhibiting exosomes in the niche [63]. In addition, miR-210 overexpression was reported to play a potential prognostic role in upper tract urothelial carcinoma [189] and oropharyngeal squamous cell carcinomas [190].

In addition, the expression of circFAM120A was significantly downregulated in both hypoxic lung adenocarcinoma cells and cancer tissue from patients with lymph node metastasis, implying its potential to be a new biomarker of lung adenocarcinoma hypoxia [28]. Moreover, circRNAs lack a 5’ cap and 3’ ends, endowing them with more stable properties than parent linear RNAs [191]. Together with their abundant and conserved characteristics, these properties make circRNAs a remarkable candidate biomarker for neoplastic diseases.

### Potential clinical utility of regulatory mechanisms shared between HIF-1α and ncRNAs

The current practical applications related to regulatory mechanisms shared between HIF-1α and ncRNAs are relatively scarce but inspiring. For instance, most clear cell renal cell carcinomas are marked by the loss of VHL tumor suppressor gene function, continuous expression of HIF-1/2α, and maladjusted expression of oncogenic miRNAs. Rustum *et al.* found that the levels of specific biomarkers associated with drug resistance in clear cell renal cell carcinoma, such as HIFs, oncogenic miR-155 and miR-210, and VEGF, could be selectively downregulated by methylselenocysteine or seleno-L-methionine in a dose- and time-dependent manner, which conferred existing anticancer therapies with enhanced therapeutic efficacy and selectivity [192]. Similarly, the antitumor effect of a novel synthetic derivative of curcumin treatment seen in pancreatic cancer was partially attributed to its inhibition of the expression of miR-21, miR-210, and HIF-1α, which are aberrantly upregulated under hypoxic conditions [193]. Additionally, Isanejad *et al.* reported that combination hormone therapy with 5-week interval exercise training could inhibit tumor angiogenesis in a mouse model of breast cancer, and the underlying mechanism could be partially explained by the suppressive effect of this combination therapy on the miR-21/HIF-1α signaling pathway [194]. Xu *et al.* suggested that targeting carcinostatic miR-338-3p/HIF-1α axis was conducive to sensitizing hepatocarcinoma cells to sorafenib [102], and Bertozzi *et al.* found that miR-17-5p and miR-155 were involved in camptothecin-induced HIF-1α reduction in human cancer cells due to their specific targeting of HIF-1α mRNA [195].

Encouragingly, ncRNAs have been increasingly considered as potential cancer therapeutic targets owing to their tissue specificity, high expression levels and crucial roles in tumor growth and progression. To date, the development of RNA-targeting methods has provided tremendous opportunities to modulate ncRNAs for cancer therapy [196, 197]. Most excitingly, novel classes of RNA-based therapeutics show great potential to modulate ncRNA activity in diverse ways [198]. Although most ncRNA-targeted treatments remain in the early stages of development, future technical innovations will provide new opportunities, and better insights into the associations between HIF-1α and ncRNAs in cancer biology will lay wide theoretical foundations for ncRNA-related targeted therapies.

## Conclusions

Continuing evidence indicates that both HIF-1α and ncRNAs play essential roles in human cancers. In this review, we have described the reciprocal regulation between HIF-1α and ncRNAs in terms of transcription, translation, and protein stability, as well as their effects on the various biological behaviors of tumor cells. We also evaluated the prospective HRN biomarkers with potential for the diagnosis and prognosis of cancer, as well as the potential clinical applications related to the regulatory mechanisms shared between HIF-1α and ncRNAs in cancer treatment. Given the large number of lncRNAs and the intense research efforts to identify and evaluate these genes, a large number of lncRNAs surely remain to be further discovered. It is certain that an improved understanding of the interplay between HIF-1α and ncRNAs will provide useful insights into tumorigenicity and may lead to novel clinical applications.

## Data Availability

Not applicable

## Abbreviations

3’-UTR: 3’-untranslated region
Bcl-2: B-cell CLL/lymphoma 2
ceRNAs: Competing endogenous RNAs
circRNAs: Circular RNAs
CREB: CAMP responsive element binding protein 1
DNM2: Dynamin 2
EGLN1: Egl-9 family hypoxia-inducible factor 1
EMT: Epithelial-mesenchymal transition
EPHB4: EPH receptor B4
FIH1: Hypoxia-inducible factor 1, alpha subunit inhibitor
GPD1L: Glycerol-3-phosphate dehydrogenase 1-like
HDACs: Histone deacetylases
HIF-1α: Hypoxia-inducible factor-1 alpha
HOXA9: Homeobox A9
HRCs: Hypoxia-responsive circRNAs
HREs: Hypoxia-response elements
HRLs: Hypoxia-responsive lncRNAs
HRMs: Hypoxia-responsive miRNAs
HRNs: Hypoxia-responsive ncRNAs
IDH2: Isocitrate dehydrogenase 2
ILF3: Interleukin enhancer binding factor 3
IPO7: Importin 7
IRS1: Insulin receptor substrate 1
KDM1B: Lysine (K)-specific demethylase 1B
lncRNAs: Long ncRNAs
MIIP: Migration and invasion inhibitory protein, OS: overall survival
miRNAs: MicroRNAs
ncRNAs: Noncoding RNAs
NDRG1: N-myc downstream regulated gene 1
OS9: Osteosarcoma amplified 9
PHDs: Prolyl hydroxylase domain enzymes
PHLPP1: PH domain and leucine rich repeat protein phosphatase 1
PKM2: Pyruvate kinase 2
pri-miRNAs: Primary miRNAs
PRMT9: Protein arginine methyltransferase 9
PTEN: Phosphatase and tensin homolog
PTPN1: Protein tyrosine phosphatase, non-receptor type 1
RASSF8: Ras association domain family member 8
RPS6KB1: Ribosomal protein S6 kinase, polypeptide 1
SDHD: Succinate dehydrogenase complex, subunit D
SLC7A5: Solute carrier family 7, member 5
SNAIL: Snail family zinc finger 1
VASP: Vasodilator-stimulated phosphoprotein
VHL: Von Hippel Lindau
WSB1: WD repeat and SOCS box containing 1
