# Correction to: The interplay between HIF-1α and noncoding RNAs in cancer

**DOI:** 10.1186/s13046-020-01544-8

**Published:** 2020-03-03

**Authors:** Xiafeng Peng, Han Gao, Rui Xu, Huiyu Wang, Jie Mei, Chaoying Liu

**Affiliations:** 1 Department of Oncology, Wuxi People’s Hospital Affiliated to Nanjing Medical University, 299 Qingyang Road, Wuxi, 214023 China; 2 The First Clinical Medicine School, Nanjing Medical University, Nanjing, 211166 China; 3 Wuxi School of Medicine, Jiangnan University, Wuxi, 214122 China; 4 School of Basic Medical Sciences, Nanjing Medical University, Nanjing, 211166 China

**Correction to: J Exp Clin Cancer Res**

**https://doi.org/10.1186/s13046-020-1535-y**

In the original publication of this manuscript [1], Fig. 2 contains incorrect labels and feedback loops. The revised version of Fig. 2 is shown below.

**Fig. 2:**
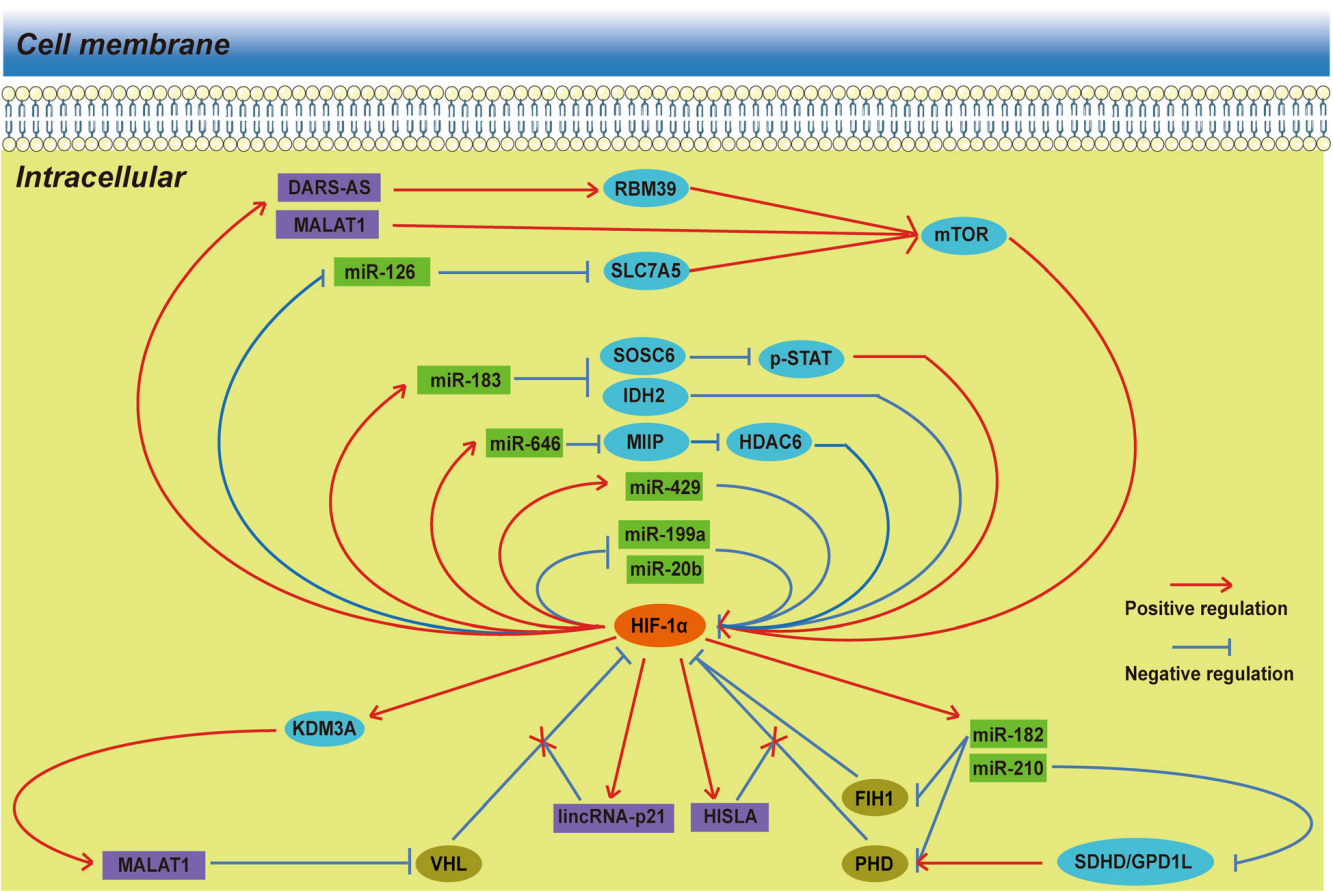
Reciprocal feedback loops between HIF-1α and ncRNAs. In addition to a unidirectional regulation pattern, there are several direct or indirect feedback loops between HIF-1α and ncRNAs. It seems quite feasible that the ncRNAs, HIF-1α and other co-operators would eventually intertwine to form mutually reciprocal feedback loops in both positive and negative manners. In addition to common feedback loops, lincRNA-p21 and HISLA can block VHL- and PHD-dependent HIF-1α repression instead of directly interacting with HIF-1α and other co-operators

In the first paragraph of the ‘Negative feedback loop between HIF-1α and ncRNA’ section, there are two instances of ‘miR-439’; these should instead read ‘miR-429’.
